# Hypoxia Imaging Endoscopy Equipped with Laser Light Source from Preclinical Live Animal Study to First-In-Human Subject Research

**DOI:** 10.1371/journal.pone.0099055

**Published:** 2014-06-10

**Authors:** Kazuhiro Kaneko, Hiroshi Yamaguchi, Takaaki Saito, Tomonori Yano, Yasuhiro Oono, Hiroaki Ikematsu, Shogo Nomura, Akihiro Sato, Motohiro Kojima, Hiroyasu Esumi, Atsushi Ochiai

**Affiliations:** 1 Department of Gastroenterology, Endoscopy Division, National Cancer Center Hospital East, Kashiwa, Chiba, Japan; 2 Division of Science and Technology for Endoscopy and Surgery, National Cancer Center Hospital East, Kashiwa, Chiba, Japan; 3 Imaging Technology Center, FUJIFILM Corporation, Kaisei, Kanagawa, Japan; 4 Clinical Trial Section, National Cancer Center, Kashiwa, Chiba, Japan; 5 Department of Pathology, National Cancer Center Hospital East, Kashiwa, Chiba, Japan; 6 Research Institute for Biomedical Sciences, Tokyo University of Science, Noda, Chiba, Japan; National Cancer Institute, National Institutes of Health, United States of America

## Abstract

A goal in next-generation endoscopy is to develop functional imaging techniques to open up new opportunities for cancer diagnosis. Although spatial and temporal information on hypoxia is crucial for understanding cancer physiology and expected to be useful for cancer diagnosis, existing techniques using fluorescent indicators have limitations due to low spatial resolution and invasive administration. To overcome these problems, we developed an imaging technology based on hemoglobin oxygen saturation in both the tumor and surrounding mucosa using a laser endoscope system, and conducted the first human subject research for patients with aero-digestive tract cancer. The oxygen saturation map overlapped the images of cancerous lesions and indicated highly heterogeneous features of oxygen supply in the tumor. The hypoxic region of the tumor surface was found in both early cancer and cancer precursors. This technology illustrates a novel aspect of cancer biology as a potential biomarker and can be widely utilized in cancer diagnosis.

## Introduction

The cancer microenvironment is highly heterogeneous and hypoxia is strongly associated with the biological features of cancer [1]–[7]. Moreover, increasing evidence suggests that hypoxia is a critical component of cancer stem cell niche [8]. Thus, examinations into cancer hypoxia have been performed [9]–[11], but measurements having sufficient spatial-temporal resolution remain to be established.

Endoscopy is a suitable method for directly accessing the inside of the body and observing the cancerous lesion at high resolution. Therefore, an endoscope that can visualize the cancer microenvironment will open up new opportunities for cancer diagnosis and biological studies.

However, current methods, including fluorescent labelling techniques [12], [13] and hemoglobin absorption-based techniques [14]–[16], are limited in their applications to endoscope systems. In fluorescent labelling techniques, the spatial distribution of fluorescence is blurred because of a lack in specificity, low target accumulation, prolonged high retention and background, although improvements in agents continue to be made. In hemoglobin absorption-based techniques, many spectral images are required to detect the spectral differences between oxy- and deoxy-hemoglobin. Capturing variable wavelength images is time-consuming and the results are often blurred because the target does not remain in a state of rest under endoscopic observation.

## Results

### Development of hypoxia imaging technology

Herein, we developed an imaging technology that can derive the oxygen saturation (StO_2_) images from small numbers of wavelength measurements. There were two challenges in deriving the StO_2_ of the tissue in alimentary tracts from the differences in absorption spectra between oxy- and deoxy-hemoglobin using small numbers of wavelengths. First, the difference in optical absorption spectra in visible light region is small and the bandwidth between isosbestic points is very narrow. Second, reflectance of a tissue depends on hematocrit (Hct) as well as StO_2_, because light absorption increases according to increases in hemoglobin density.

**Figure 1:**
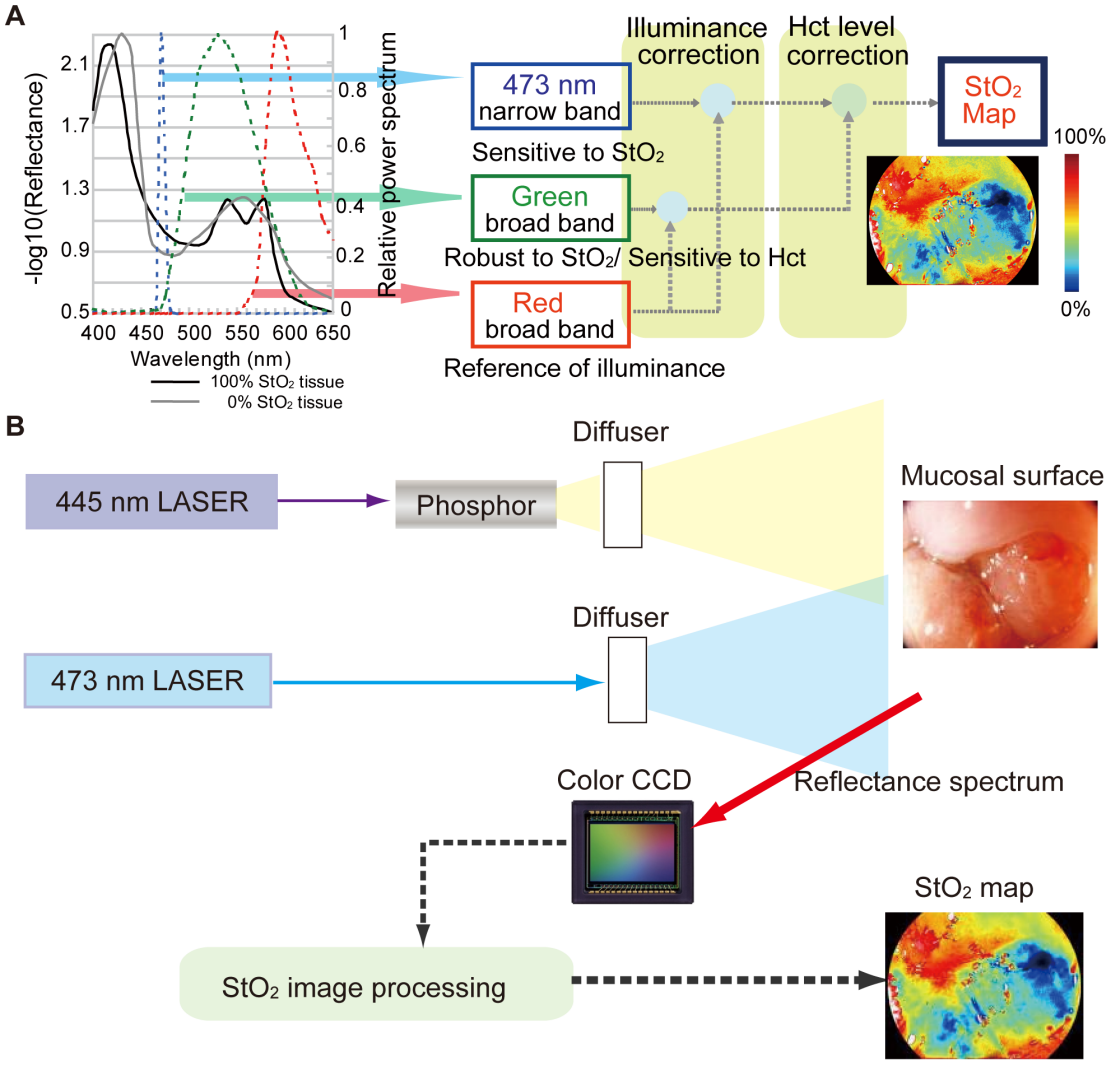
Mechanism of hemoglobin oxygen saturation imaging and schematic illustration of prototype endoscope system. (A) Illustration of the mechanism (see text for details.) (B) The 445-nm laser excited a phosphor equipped at the tip of the endoscope and emitted white light. The 473-nm laser light was emitted without the phosphor excitation. These two lights alternately illuminated the mucosal surface and the reflected lights were sequentially detected with a colour CCD in synchronization with light switching. The obtained images were processed and transformed into a StO_2_ map.

Selection of wavelength and bandwidth is vitally important in deriving StO_2_ from small numbers of wavelength images. We found that a combination of narrow and two broad-band spectra are most suitable for derive StO_2_ in alimentary tract tissue. (i) A narrow band at 473 nm was selected to detect the variations in StO_2_, while the change in reflectance according to the variance in StO_2_ at 473 nm is largest in the visible light region. We made the bandwidth very narrow (a few nanometres in width) to prevent a decrease in StO_2_ sensitivity caused by wavelength width across the isosbestic points. (ii) A green broad-band was selected to detect variations in Hct. The green band is sensitive to variations in Hct because the absorption coefficient of hemoglobin is large. To make the signal robust to variations in StO_2_, we broadened the green band (500–600 nm) across a few isosbestic points. (iii) A red broad-band was selected to detect the changes in illumination caused by the distance between the light exit window of the endoscope and the illuminated tissue. The red broad-band (600–700 nm) is robust to variations in StO_2_ and Hct because the absorption coefficient of hemoglobin is small. We applied a computational technique to derive StO_2_ signals from these three light signals. Briefly, the 473 nm signal and the green band signal are normalized against the red signal to cancel their dependence on illuminance. The normalized 473 nm signal is calibrated using the normalized green signal to cancel its dependence on Hct. Thus, pure StO_2_ information can be obtained (Fig. 1A).

Based on these strategies, we developed an imaging system equipped with laser diodes of 445 and 473 nm and a white fluorescent pigment body. Broad-band diffused light is emitted from the white fluorescent pigment body excited by the 445-nm laser diode. The 473-nm diffused light, which is useful for illuminating narrow band light for StO_2_ signals, is emitted by switching the laser light source from 445 nm to 473 nm. A color CCD sensor captures the 473-nm narrow band, and the green and red broad-band images (Fig. 1B).

**Figure 2:**
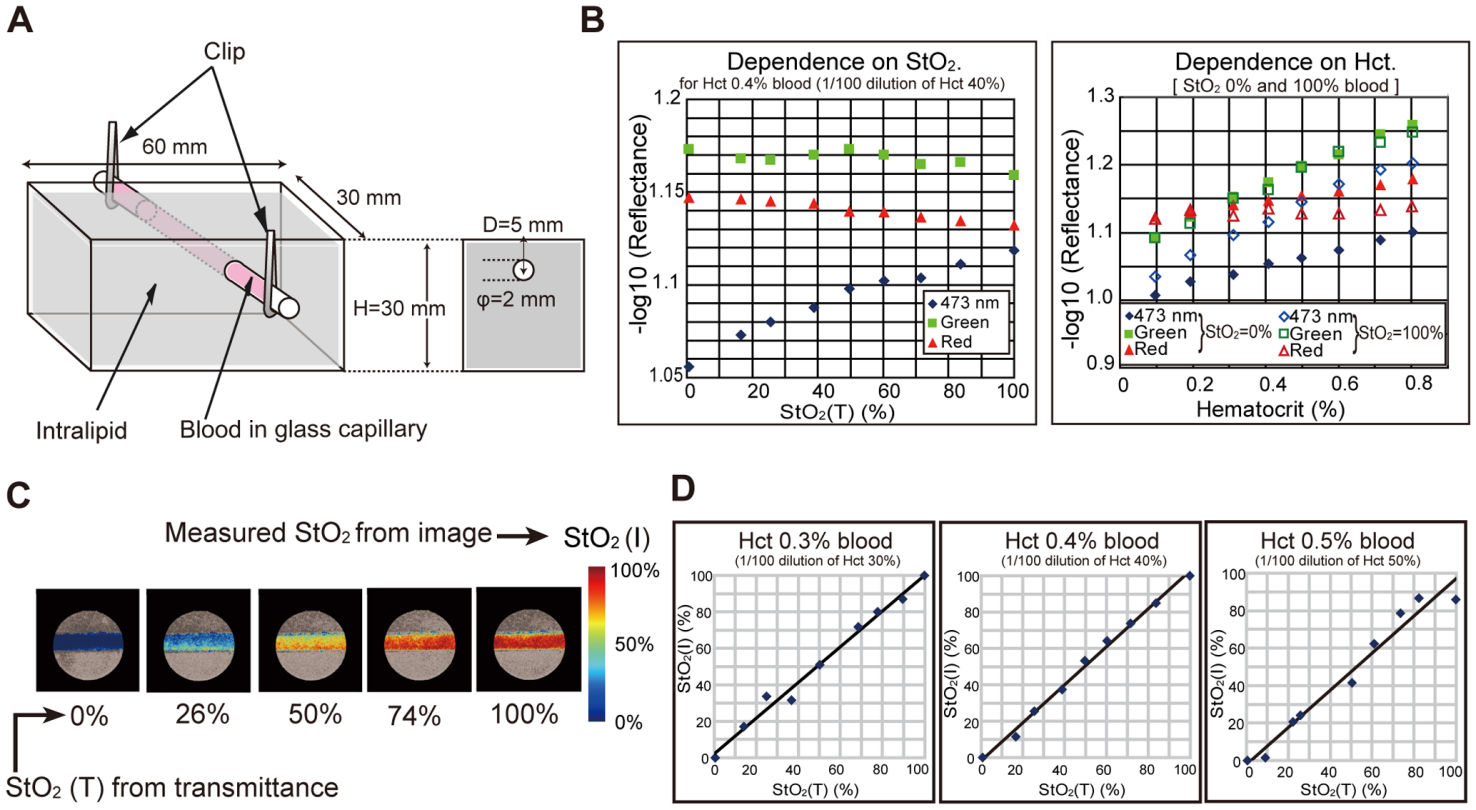
Verification of hemoglobin oxygen saturation imaging by observing a phantom. (A) Blood vessel phantom consisted of a glass tube filled with diluted blood and aqueous solution of intralipid. The intralipid solution strongly scattered incident light to simulate the living tissue around blood vessel. (B) The observed optical densities of the blood vessel at the three bands were dependent on StO2(T) (left) and Hct (right). Here, StO2(T) denotes the supposedly correct value of StO2 derived by analyzing the transmittance spectrum of blood. (C) StO2(I) map (derived by image processing) of the vessel. (D) Comparison of StO2(I) with StO2(T) (derived by measurement of transmittance spectra).

In order to clarify the changes in quantity of light according to StO_2_ and Hct, we introduced an intralipid phantom consisting of a glass capillary containing blood (Fig. 2A). The quantities of light as a function of StO_2_ and Hct for 473 nm in the narrow, broad green and red bands were captured by the imaging system. Figure 2B shows that the 473 nm signal is sensitive to the change in StO2 and the green and red broadband signals are robust to that. Images were processed to create hemoglobin saturation maps. Figure 2C shows representative hemoglobin saturation pseudocolor maps of blood for different StO_2_ levels. Measurements of hemoglobin saturation for different Hct levels derived with the imaging system corresponded well with the StO_2_ measured using a spectral meter (Fig. 2D).

### In vivo imaging of nude mouse transplanted with cancer cells

**Figure 3:**
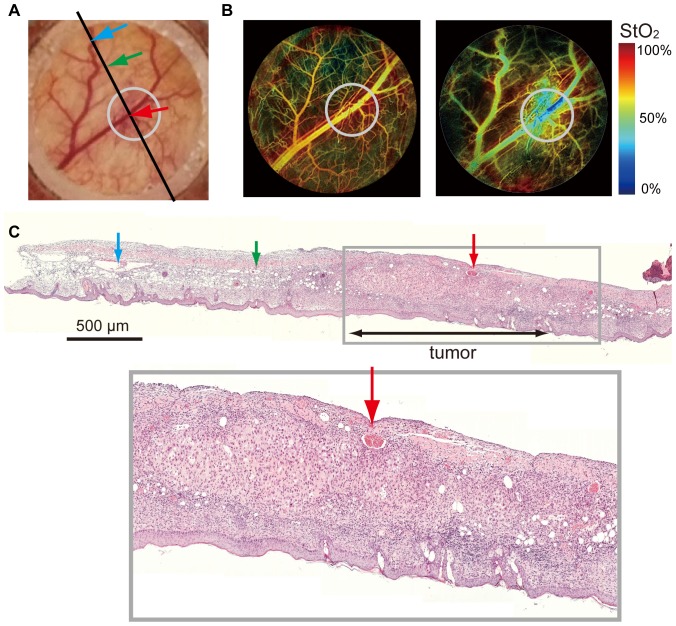
*In vivo* imaging of nude mouse implanted with cancer cells. (A) White light image of the mouse. The solid line corresponds to the cross-section of pathological assessment. (B) StO2 map of mouse before transplantation (left). Hypoxia developed at the tumor at 7 days after transplantation (right). (C) Histological picture (hematoxylin-eosin stained) of skin resected from the mouse at 14 days after transplantation (upper right, lower). Arrows indicate corresponding vessels.

We then examined our approach using animal models. We used nude mice transplanted with A549 human cancer cells and attached window chambers [17] to the skin-peeled area, which kept the skin extended and enabled us to observe blood vessels under the skin. A549 cells were transplanted under the skin in the chambers (Fig. 3A). Three-band wavelength images using the imaging system to derive the StO_2_ map from spectroscopic data were obtained (Fig. 3B left). The StO_2_ map at 7 days after transplantation showed that the low StO_2_ area was merged with the tumor-injected region where the tumor mass and aberrant tumor angiogenesis were augmented (Fig. 3B right). We confirmed the presence of cancer cells in histological images (Fig. 3C).

### In vivo imaging of alimentary tracts with pigs

**Figure 4:**
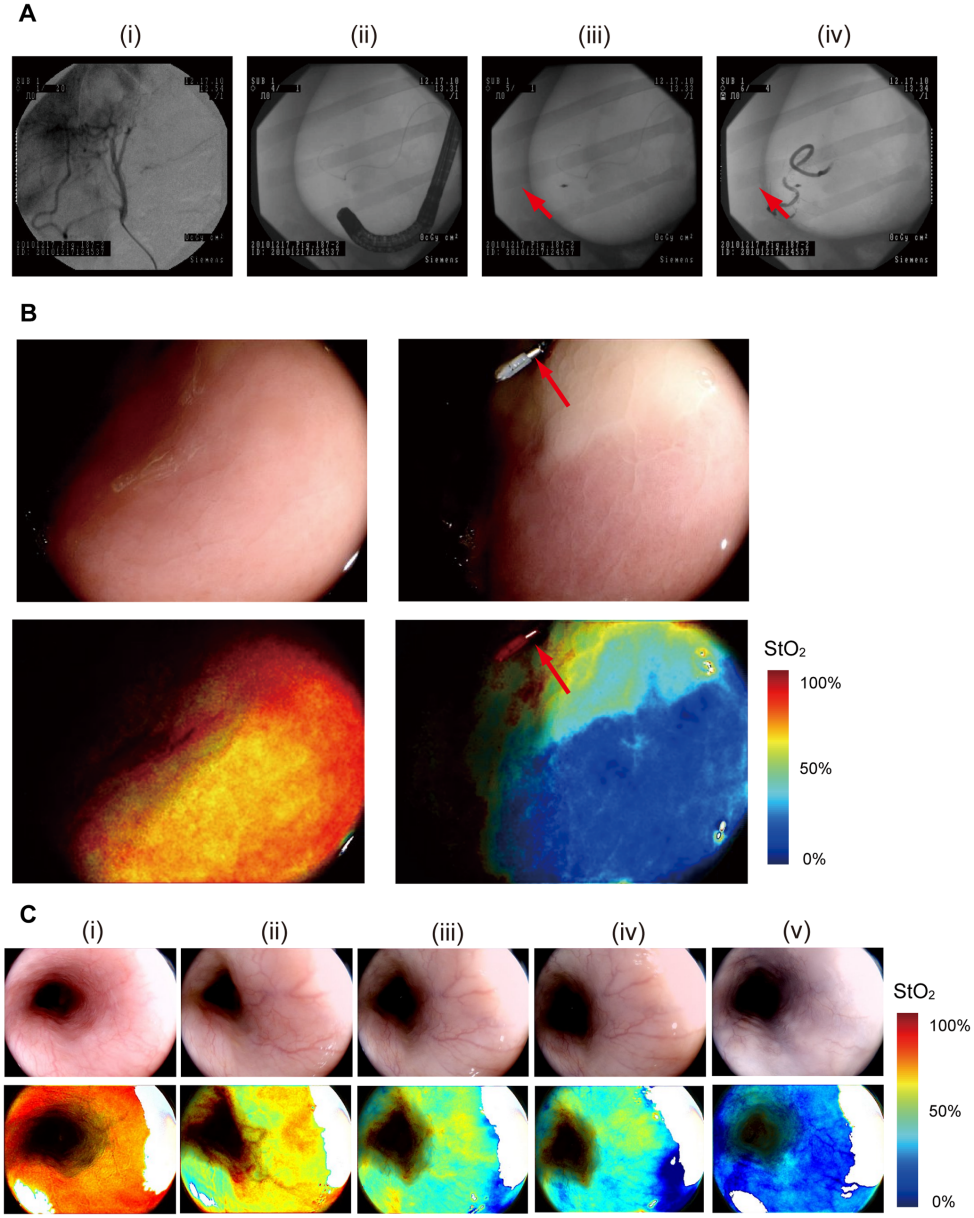
*In vivo* imaging with pig. (A) X-ray fluoroscopic images during application of transcatheter arterial embolization in pig stomach. (i) Image of the target vessel. (ii) Image of the endoscope inserted into the stomach. (iii) Red arrow indicates marking clip to identify the target area. (iv) Image of injected hystoacryl medium into the artery from the catheter. (B) White light images (upper) and StO_2_ maps (lower) of the gastric mucosal surface visualized by laser endoscope system before embolisation (left) and five minutes after embolisation (right). (C) White light images (upper) and StO_2_ maps (lower) of the esophagus tissue (i) before the stomach removal, (ii) after the stomach removal, (iii) two minutes after the KCl injection, (iv) four minutes after the KCl injection and (v) twenty minutes after the KCl injection.

We then conducted an *in vivo* imaging experiment in pigs with a hypoxic area in the stomach tissue generated by transcatheter arterial embolization. Figure 4A shows a series of fluoroscopic images from the injection of the agent to embolization. Before embolization, high StO_2_ value was observed (Fig. 4B lower left). Five minutes after embolization, the StO_2_ map showed emergence of a hypoxic area corresponding to artificial vessel occlusion (Fig. 4B lower right). We also observed the change in StO2 at the esophagus. The StO2 was initially normal (around 70%) (Fig. 4C(i)). After the stomach removal, it declined to around 50–60% (Fig. 4C (ii)). The StO2 further dropped to near zero after the KCl injection (Fig. 4C (v)).

### Human subject research

**Figure 5:**
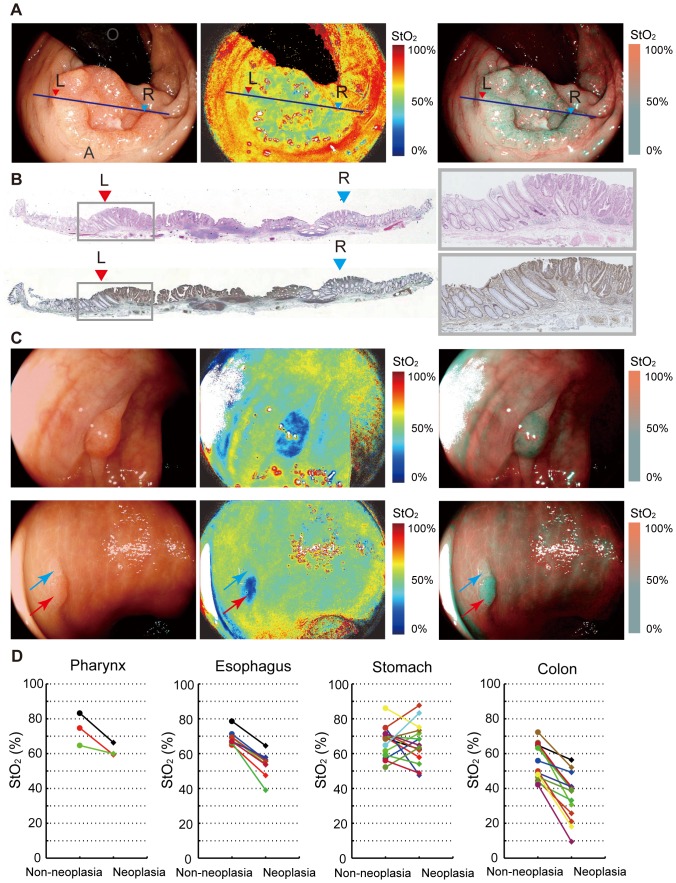
StO_2_ maps obtained in human subject research. (A) White light image by endoscopic observation in rectal adenocarcinoma (left). Line (L-R) corresponds to cross-section of pathological diagnosis. StO_2_ map visualized by laser endoscope system (middle: pseudocolor StO_2_ image; right: StO_2_ overlay image). (B) Cross-section appearance stained with H&E (upper) and HIF1 alpha antibody (lower) corresponding to the hypoxic area visualized with StO_2_ map. (C) Endoscopic images of a colorectal adenoma (upper) showing clear hypoxia: white light image (upper left), pseudocolor StO_2_ map (upper middle) and overlayed image (upper right). Another case of a colonic lesion (lower) consisting of an adenoma (red arrow) and a hyperplasia (blue arrow): white light image (lower left), pseudocolor StO_2_ map (lower middle) and overlayed image (lower right). Only the adenoma was detected as hypoxia. (D) Observed StO_2_ differences between neoplastic and non-neoplastic areas: For comparing pathology specimens and endoscope images, the line on the endoscopic image corresponding to the cross-section was determined. StO_2_ levels at neoplasic and non-neoplasic areas along this line were then calculated using this StO_2_ map.

Next, we conducted a proof-of-the-concept research [18] for 40 patients with neoplastic lesions in the esophagus including the pharynx, stomach and colorectum (Table 1). In this first in human subject research (UMIN 000004983), two types of StO_2_ images were used. One was a pseudocolor StO_2_ image that showed StO_2_ levels as different hues, and the other was a StO_2_ overlay image that overlapped StO_2_ levels in blue on a white light illumination image to detect background mucosa. Figure 5A shows an example of the StO_2_ map for rectal adenocarcinoma. The hypoxic area was completely visible on the StO_2_ map corresponding to the cancer region. Pathological diagnosis by H&E (hematoxylin and eosin) staining showed adenocarcinoma infiltrating into the submucosal layer (Fig. 5B upper). In this case, HIF1 alpha expression in immunohistochemical staining was found in the area described as hypoxic on the StO_2_ map (Fig. 5B lower).

**Table 1:** Patients Characteristics.

(n = 40)	
Age	
Mean (y)	70.8
Range (y)	49–85
Gender n (%)	
Male	32 (80%)
Female	8 (20%)
Location n (%)	
Pharynx	3 (7%)
Esophagus	8 (20%)
Stomach	15 (38%)
Colorectum	14 (35%)

The hypoxic area was confirmed in early cancer. Eight colorectal adenomas with histological low-grade atypia were also detected as hypoxia, ranging between 3 mm and 10 mm in diameter (Fig. 5C upper, Video S1). Furthermore, one of the eight adenomas showed co-lesions combined with adenoma and hyperplasia. The low StO_2_ region was detected in the adenoma portion, but not in the hyperplasic portion (Fig. 5C lower).

Figure 5D shows the observed StO_2_ differences between neoplastic and non-neoplastic areas. Median StO_2_ differences between neoplastic and non-neoplastic areas in the pharynx, esophagus, stomach and colorectum were −15.4%, −14.5%, −5.1% and −21.5%, respectively. There were significant differences in StO_2_ levels between neoplastic and non-neoplastic areas in the esophagus (p = 0.0078 on Wilcoxon signed-rank test, 8 patients in each area (n = 8)) and colorectum (p = 0.0001, n = 14), but not in the stomach (p = 0.9341, n = 15) or pharynx (p = 0.2500, n = 3). Furthermore, sensitivity of neoplasia, defined as the proportion having correctly detected neoplasia, in the pharynx, esophagus, stomach, and colorectum was 67%, 100%, 33% and 86%, respectively.

## Discussion

This is the first human subject research using the endoscopic hemoglobin oxygen saturation imaging technology for patients with aero-digestive tract cancers or adenomas. Before the human subject research, we evaluated our technology using a phantom and animals.

From the results of the phantom experiments, we confirmed that the green signal is useful for the separation of StO_2_ and Hct information from the 473 signal. The characteristic that the red signal is robust to Hct and StO_2_ is also important for the endoscopic application. The movement of the endoscope and alimentary tract tissue causes the change in illuminance during endoscopic observations. The robustness of red signal to Hct and StO_2_ enables us to correct the change in illuminance.

Using dorsal skin-fold chamber mouse model, we detected temporal and spatial heterogeneity of oxygen saturation in tumor region. In a previous study [16], similar results were obtained using a hyperspectaral imaging system. The hyperspectral imaging system acquired images from 500 to 575 nm in 5-nm intervals to derive StO_2_ map. We consider the hyperspectral imaging system needs long imaging times and a high-power source for sample illumination. Advantages of our technology in endoscopic application are short imaging times and the simplicity of the instruments. In *in-vivo* imaging experiments with pigs, we confirmed that our hypoxia imaging technology with endoscope can visualize the StO_2_ map of alimentary tract tissue in real-time.

From the results of our human subject research, we can see that hypoxic imaging could clearly distinguish neoplasia from non-neoplasia in the esophagus and colorectum. As clinical benefits, screening of oesophageal and colorectal neoplasia, or prediction to efficacy of chemotherapy or radiotherapy can be utilized. Compared to the esophagus and colorectum, gastric cancer showed variations in tumor oxygen levels. Some cases exhibited hyperoxic conditions when compared with noncancerous areas around gastric cancer, but no significant differences were observed in clinicopathological findings between hypoxic cases and non-hypoxic including hyperoxic cases (Table 2). Further examination of organ specific hypoxia will be required.

**Table 2:** Clinicopathological findings of the study with gastric cancer patients.

		Hypoxia	Non-hypoxia
		(n = 9)	(n = 6)
Age (y)	≧ 70 (n = 9)	5 (56%)	4 (44%)
	<70 (n = 6)	4 (67%)	2 (33%)
Gender	Male (n = 11)	6 (55%)	5 (45%)
	Female (n = 4)	3 (75%)	1 (25%)
Histologic type	Dif. (n = 12)	7 (58%)	5 (42%)
	Undif. (n = 3)	2 (67%)	1 (33%)
Macroscopic type	Elevated (n = 4)	3 (75%)	1 (25%)
	Depressed (n = 11)	6 (55%)	5 (45%)
Size (cm)	≧ 2 (n = 7)	5 (71%)	2 (29%)
	<2 (n = 8)	4 (50%)	4 (50%)
Location	U (n = 1)	1 (100%)	0 (0%)
	M (n = 10)	5 (50%)	5 (50%)
	L (n = 4)	3 (75%)	1 (25%)

Our endoscopic hypoxia imaging method provides a better opportunity to investigate the characteristics of cancer hypoxia. Some techniques detecting hypoxia by capillary methods, molecular biologic analysis and immunohistochemistry using histologic specimens have been reported. In the capillary method and molecular biological analysis [19], hypoxic conditions can be observed in only limited parts of the whole tumor, and features of whole tumors with hypoxia cannot be visualized. On immunohistochemistry using histologic specimens, visualization in real time is impossible. However, hypoxia imaging using this system is superior in visualizing the hypoxic conditions of whole tumors both sequentially and in real-time. No other method can simultaneously monitor oxygen concentrations in both cancerous and noncancerous areas.

Our data strongly suggest that the microenvironment of oxygen supply to the tumor is spatially and temporally heterogeneous. This endoscope system enables us to observe spatial and temporal information of hypoxic conditions in human tumors. Moreover, we can directly acquire human cancer cells under various hypoxic conditions from biopsy samples. From these features, this endoscope system is expected to contribute to research into cancer biology, as well as into medications and treatment methods based on cancer hypoxia.

## Methods

### Ethics statement

This human subject research was approved by the National Cancer Center Hospital East Institutional Review Board (K23-2). A written informed consent was obtained from each patient. Animal experiments were approved by the Animal Ethics Committee of the National Cancer Center Hospital East (K13-015). We used horse blood, which was purchased from Nippon Bio-test Laboratories Inc., in the phantom experiment. The animal work that produced the blood samples was approved by the Animal Ethics Committee of the Nippon Bio-test Laboratories Inc.

### Laser endoscope system

We developed a prototype laser endoscope system composed of two types of laser and a white fluorescent pigment body (20 mW, 473 nm laser diode, and 1 W, 445 nm blue laser diode; Nichia, Japan) as light sources and a commercial endoscope system (EG-590ZW gastroscope, and EC-590ZW3 colonoscope; Fujifilm, Japan).

### Phantom

We created a vessel phantom composed of a glass microcapillary, Intralipid-10% (fat emulsion) and blood (horse blood, stored in an equal volume of Alsever's solution), inside a container of dimensions 60 mm×30 mm×30 mm (Fig. 2A). The vessel phantom imitates typical human tissue at a 100× magnified scale. The inside diameter of the glass microcapillary is 2 mm. The depth of glass microcapillary from the liquid surface is 5 mm. Thus, the vessel phantom imitates the 20-µm diameter vessel in human tissue at 50 µm depth. The size of the vessel is 100× magnified; therefore the scattering coefficient of Intralipid and absorption coefficient of blood is one hundredth that of typical human tissues. Blood is diluted with pure water so that the hematocrit value is 0.5%, 0.4% or 0.3%. Sodium hydrosulphite (Na_2_O_4_S_2_) is used for de-oxygenation of blood. Transmittance spectra are measured with a spectral radiometer (TOPCON SR-UL1) and a Xenon light source, and the true value of oxygen saturation is calculated from transmittance spectra.

### Dorsal skin-fold chamber mouse model

We anesthetized each mouse by intraperitoneal injection of 0.4 ml avertin (1.2 wt% 2,2,2-tribromoethanol and 1 wt% 2-propanol dissolved in saline). This treatment maintained the mouse under anethesia for about 30 min. To minimize pain, the following operation of mounting a window chamber was conducted within 15 min. We peeled a small part of the dorsal skin and then attached a custom-made skin-fold window chamber that kept the subcutaneous blood vessels in the skin-peeled area observable. The circular glass window was 9 mm in diameter. We prepared a suspension of A549 human lung cancer cells by mixing 2×10^5^ cells with 40 µl of BD Matrigel™ and injected it under the skin in the chamber. After the experiment, all the mice were sacrificed by deep euthanasia using diethyl ether.

### Cell culture

A549 human lung cancer cells (ATCC) were cultured in Dulbecco's modified Eagle medium (DMEM) at 37°C.

### In vivo imaging of alimentary tracts with pigs

We used two conventional female Large White and Duroc pigs (40 kg) bred in a closed colony. All efforts were made to minimize animal suffering. We anesthetized the pigs by firstly administration of 20 ml ketalar (500 mg/10 ml) intramuscularly and 250 mg isozol intravenously, and then inhalation of sevoflurane after intubation. Anesthesia was maintained via a circular breathing system. All the following procedures were carried out under the anesthesia. Under X-ray guidance with a fluoroscopic system (Powermobile C-ARM Angiographic System; Siemens), an angiographic catheter (Selecon safe tip; Terumo, Japan) was placed into the common hepatic artery, and a microcatheter (Progreat alpha; Terumo) was placed into the left gastroepiploic artery. We used Histacryl (B. Braun Biosurgicals) diluted 10-fold with Lipiodol (Terumo) as the embolic agent. We injected the agent with the micro catheter to embolize the arteries connected to the stomach. This embolization made the stomach partially hypoxic. The StO_2_ map of the stomach was observed with the hemoglobin oxygen saturation imaging system. After the observation of the stomach, we opened the abdomen and removed the stomach for a histological evaluation. We also placed the endoscope at the esophagus and observed the change of the pig's oxygenation state before the stomach observation, after the stomach removal and after an intravenous administration of potassium chloride (KCl) to cause cardiac arrest.

### Human subject research

Patients who had been confirmed to have pharyngeal, oesophageal, gastric, or colorectal neoplasia by previous endoscopic examination were enrolled. Eligibility criteria were as follows: age of 20 years or more; and male or female patients. After conventional endoscopy was performed, hypoxia imaging was observed using prototype endoscopy. To compare histologic findings to hypoxia imaging, all patients received endoscopic treatment, such as polypectomy, EMR or ESD after conventional and hypoxia imaging endoscopy. When comparing pathological findings, we determined the corresponding areas of neoplasia and non-neoplasia in the endoscope images and obtained StO_2_ levels from the StO_2_ map.

### Pathological assessment

We performed immunohistochemical expression of HIF-1 alpha, which accumulates under hypoxia^5^, to evaluate hypoxic status on the histological slide. Sections (5 µm) from paraffin-embedded slices that included the most representative area were selected and used for immunohistochemical staining of HIF-1 alpha (Rabbit polyclonal antibody to HIF-1 alpha(ab104072); Abcam, Tokyo, Japan). Citrate buffer (pH 6.0) was used for antigen retrieval, and antigen dilution was ×100. Human lung adenocarcinoma and squamous cell carcinoma were used as positive controls.

### Statistical analysis

StO_2_ levels were measured in both neoplastic and non-neoplastic areas for each patient. Stratified by cancer region, this paired data was compared using Wilcoxon signed-rank test. Among gastric cancer patients, we summarised the patient characteristics according to the remainder calculated by subtracting StO_2_ levels of the normal tissue from those of tumor tissue, as in some cases, tumor tissue appeared as non-blue color (same color as normal tissues). All P values were two-sided.

## Supporting Information

Video S1 **Endoscopic video image showing the hypoxic feature of a colorectal adenoma in real time: white light image (left upper), pseudocolor StO_2_ map (left lower) and overlayed image (right).**
(ZIP)
